# Treatment-seeking behaviour among 15–49-year-olds with self-reported heart disease, cancer, chronic respiratory disease, and diabetes: a national cross-sectional study in India

**DOI:** 10.1186/s12889-023-17123-3

**Published:** 2023-11-08

**Authors:** Fredh Netterström-Wedin, Koustuv Dalal

**Affiliations:** 1 Division of Public Health Science, School of Health Sciences, Mid Sweden University, Sundsvall, Sweden; 2 School of Public Health and Community Medicine, University of Gothenburg, Gothenburg, Sweden

**Keywords:** Chronic disease, Delivery of health care, Health care seeking behavior, Health services research, Non-communicable diseases

## Abstract

**Background:**

Eighty per cent of India´s non-communicable disease (NCD) mortality is due to four conditions: heart disease, cancer, chronic respiratory disease, and diabetes, which are primarily cause-amenable through treatment. Based on Andersen’s behavioural model of health services use, the current study aimed to identify the predisposing, enabling, and need factors associated with treatment-seeking status among people self-reporting the four main NCDs in India.

**Methods:**

Cross-sectional study using secondary data. Usual residents aged 15–49 who self-reported cancer (n = 1 056), chronic respiratory disease (n = 10 534), diabetes (n = 13 501), and/or heart disease (n = 5 861) during the fifth National Family and Health Survey (NFHS-5), 2019–21, were included. Treatment-seeking status was modelled separately for each disease using survey-adjusted multivariable logistic regression.

**Results:**

3.9% of India´s 15–49-year-old population self-reported ≥ 1 of the four main NCDs (0.1% cancer, 1.4% chronic respiratory disease, 2% diabetes, 0.8% heart disease). The percentage that had sought treatment for their condition(s) was 82%, 68%, 76%, and 74%, respectively. Greater age and having ≥ 1 of the NCDs were associated with greater odds of seeking disease-specific treatment. People in the middle or lower wealth quintiles had lower odds of seeking care than the wealthiest 20% for all conditions. Women with diabetes or chronic respiratory disease had greater odds of seeking disease-specific treatment than men. Muslims, the unmarried, and those with health insurance had greater odds of seeking cancer treatment than Hindus, the married, and the uninsured.

**Conclusion:**

Predisposing, enabling, and need factors are associated with treatment-seeking status among people reporting the four major NCDs in India, suggesting that multiple processes inform the decision to seek disease-specific care among aware cases. Successfully encouraging and enabling as many people as possible who knowingly live with major NCDs to seek treatment is likely contingent on a multi-pronged approach to healthcare policy-making. The need to improve treatment uptake through accessible healthcare is further underscored by the fact that one-fifth (cancer) to one-third (chronic respiratory disease) of 15–49-year-olds reporting a major NCD have never sought treatment despite being aware of their condition.

**Supplementary Information:**

The online version contains supplementary material available at 10.1186/s12889-023-17123-3.

## Introduction

There are concerns that the global non-communicable disease (NCD) crisis cannot be resolved unless healthcare services become accessible to all in low- and middle-income countries (LMICs) [1]. Despite NCDs historically being more common in high-income countries, the global burden has shifted, with 77% of NCD deaths now occurring in LMICs [2]. Many of these deaths are premature and can be avoided by treating those living with NCDs [3, 4]. However, most LMICs have had difficulty adapting their healthcare systems to address the shift in disease burden from infectious diseases to NCDs [5]. This has resulted in unequal access to healthcare and increased disparities in health outcomes due to individual and contextual factors such as socioeconomic status and place of residence [1]. Therefore, resolving the global NCD crisis requires not only overall improvement in physical and financial accessibility of NCD treatment in LMICs but also prioritising neglected groups within these countries [1].

India is one of the LMICs currently facing the need to adapt health services toward NCD care, as rapid economic growth has led to a significant increase in the country’s NCD burden [6]. In 2007, India transitioned from a low-income country to a lower-middle-income country [7]. At the time, NCDs comprised half of the country’s yearly deaths; this number has since increased to roughly two-thirds of all annual deaths, with an estimated six million people dying from NCDs annually in India [2]. Since India is home to approximately 1.4 billion people or 18% of the world’s population [8], these deaths contribute to three-fourths of the total NCD mortality in South Asia and almost one-fifth of all NCD deaths globally [2]. Consequently, improving the NCD situation in India by ensuring that treatment is accessible to all is crucial for the country, the region, and the world.

Four NCDs — heart disease, cancer, chronic respiratory disease, and diabetes — contribute disproportionately to India’s death toll and have been the focus of ongoing control efforts. These NCDs account for approximately 80% of all NCD deaths in India and a similar percentage of the country’s premature NCD mortality [2]. In 2012, it was projected that these diseases would cost India 3.55 trillion USD in lost savings and forgone productivity by 2030 unless action is taken [6]. To address this, the government of India has implemented several nationwide programmes to control these diseases. A key initiative is the National Programme for Prevention and Control of Cancer, Diabetes, Cardiovascular Diseases and Stroke (NPCDCS), which, besides prevention and early detection, aims to provide accessible treatment for these NCDs. Since its launch in 2010, the NPCDCS has expanded to include chronic obstructive pulmonary disease and chronic kidney disease — a common sequela of diabetes [9] — and has gone from treating NCDs in 100 districts to covering all 707 sections in India [10, 11].

Another example is the Ayushman Bharat programme launched in 2018. Although not explicitly targeted toward treating NCDs, the Ayushman Bharat has been acknowledged as an essential programme for improving India’s NCD situation [12, 13]. It aims to cover hospitalisation costs for over 100 million low-income families and improve primary healthcare capacity (including the provision of NCD treatment) by building 150 000 more health and wellness centres across India [12, 14]. Governmental initiatives such as these are crucial for achieving targets set by the World Health Organization and the United Nations related to NCD management, including 80% availability of essential NCD medicines by 2025 and a one-third reduction in premature NCD mortality by 2030 relative to 2015 [15, 16].

Despite ongoing efforts by the Indian government to control the four main NCDs, progress toward ensuring treatment availability and reducing premature mortality is lagging. Poor service preparedness and capacity, including frequent stockouts of essential NCD medicines, were found during the implementation of the NPCDCS between 2013 and 2016 in the southern state of Karnataka (which has slightly better health and development than the national average) [17]. According to more recent and nationally representative data, delivery of services for NCDs varies depending on the disease and the type of service provider. Still, it needs to be improved across the country [18]. Where and when NCD services are available, incurred treatment costs are more than double those of infectious disease treatments and disproportionately affect marginalised families, such as people with low incomes and those living in rural neighbourhoods [19]. Worryingly, India is projected not to reach the targeted reduction in premature NCD mortality by 2030 [3].

Developing a better understanding of peoples’ treatment-seeking behaviours for the four major NCDs may facilitate the planning and provision of healthcare services in India. Treatment-seeking behaviour, or healthcare-seeking behaviour more generally, can be defined as “any activity undertaken by individuals who perceive themselves to have a health problem or to be ill to find an appropriate remedy” [20] and represents an extended process usually triggered by awareness of illness culminating in accessing the formal healthcare system [21, 22]. Studies focusing on such healthcare-seeking behaviours and their associated factors are essential to inform healthcare policy [23]. Yet, evidence from India about seeking treatment for the four major NCDs is scarce. Previous research based on the fourth National Family Health Survey (NFHS-4) from 2015 to 2016 focusing on diabetes and heart disease has found that various sociodemographic and economic characteristics, such as age and wealth, are associated with people’s treatment-seeking status [24, 25]. While these studies have helped elucidate the correlates of seeking treatment for some of the major NCDs in India, a comprehensive and up-to-date account that systematically examines treatment-seeking behaviours across all four main NCDs is currently missing.

The present study, therefore, aims to address this gap in the literature by analysing data from the fifth and most recent wave of the NFHS conducted in 2019–21. The NFHS-5 provides nationally representative information on individual and household characteristics, the presence of the four main NCDs, and whether people have sought treatment for these conditions. Herein, we report the percentage who have sought treatment among aware cases and the factors associated with treatment-seeking status for each of the four main NCDs. We use the widely accepted Andersen’s Behavioural Model of Health Services Use to guide our analysis [22, 26–28]. This conceptual model is particularly well-suited for this purpose since it was developed to help explain disparities in healthcare access observed in national health surveys [29]. The behavioural model posits that the use of personal health services can be explained by predisposing, enabling, and need factors [26–28]. Predisposing factors of health service use can broadly be categorised into demographic variables that serve as biological imperatives, such as age or sex, social structure variables, which reflect the status of the individual in society (e.g., education, ethnicity) or their social networks (e.g., family size, religion), and health belief variables. Enabling factors facilitate or impede the use of personal health services and consist of the available resources in the family (e.g., household income, insurance coverage) and the community (e.g., urban-rural character). Finally, need factors are important for realising access and may constitute an evaluated (e.g., diagnoses) or perceived (e.g., self-rated function) need for health services [26–28].

The present study aims to identify the predisposing, enabling, and need factors associated with seeking treatment for the four main NCDs among aware cases in India.

## Methods

### Study design and setting

This study constitutes a secondary cross-sectional analysis of NFHS-5 [30]. NFHS-5 is a nationally representative survey funded by the Ministry of Health and Family Welfare, Government of India, and conducted by the International Institute for Population Sciences (IIPS), Mumbai. It is part of the Demographic and Health Survey (DHS) Program. It provides information related to health and family welfare from 707 districts across all 28 states and eight union territories in India. Data were collected in two phases after pretesting and training: 17 June 2019 to 30 January 2020 and 2 January 2020 to 30 April 2021. The NFHS-5 used a two-stage stratified sample. Primary sampling units (PSUs) were chosen from the 2011 census of rural and urban areas in the first stage. According to community structure and services, population size and density, and the fraction of male workers in occupations other than farming, urban neighbourhoods were distinguished from their rural equivalents [31]. PSUs, which were chosen with probability proportional to size, were census enumeration blocks in urban areas and villages in rural regions. From each PSU, 22 households were selected at random by systematic sampling. All women aged 15 to 49 who were regular occupants or had stayed overnight were invited to participate. Additionally, 15% of the families were randomly chosen for interviews with all present men aged 15 to 54 [30].

Interviews were held with 636 699 households, 724 115 women and 101 839 men during NFHS-5. The household response rate, calculated by dividing the total number of interviewed households by the total number of occupied homes, was 98%. Ninety-two per cent of eligible men and 97% of eligible women completed the individual survey [30].

### Participants

**Fig. 1:**
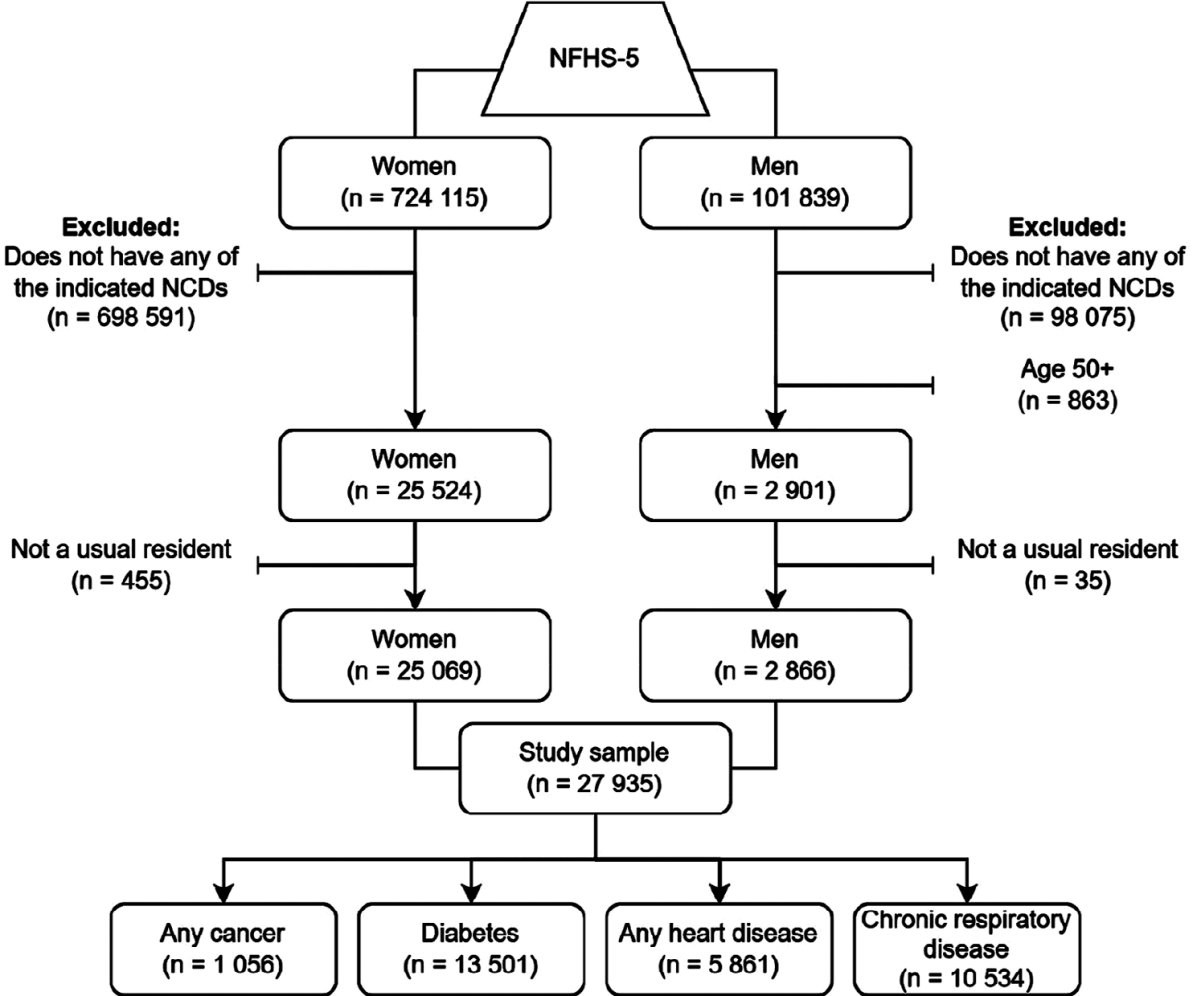
Flowchart illustrating eligibility criteria and the number of respondents included in our secondary study. The sum of cases across conditions exceeds the study sample since some respondents were multimorbid. NCDs = Non-communicable diseases NFHS-5 = National Family and Health Survey-5

Our study included 2 866 men and 25 069 women (Fig. 1). Only individuals who answered “yes” to currently having one or more NCDs were eligible: cancer, chronic respiratory disease including asthma, diabetes, and/or heart disease. Throughout this paper, we refer to these participants as aware rather than diagnosed cases since the NFHS-5 did not specifically ask about a health professional’s diagnosis. Similar to other studies using NFHS data, we excluded men aged 50 + so that both sexes had comparable age ranges [25, 32, 33]. All analyses were limited to usual (de jure) residents to avoid double-counting [34].

#### Outcome measures

The men and women who self-reported currently having cancer, chronic respiratory disease, diabetes, and/or heart disease during the interview were also asked whether they have sought treatment for their illness(es). In the NFHS-5, this was ascertained by the follow-up question: “Have you sought treatment for this problem?” whereby respondents could answer “yes” or “no”.

### Independent variables selected based on the conceptual model

We included 12 predisposing, enabling, and need factors that were available in the NFHS-5 dataset and which overlapped with the factors presented in Andersen and Newman’s [27, 28] seminal paper on the individual determinants of health service utilisation:

Predisposing factors.

- Age.
- Caste (forward or casteless, scheduled caste, scheduled tribe, other backward class).
- Years of education.
- Sex (male versus female).
- Marital status (currently married, formerly married, never married).
- Number of household members.
- Religion (Hindu, Muslim, other).

Enabling factors.

- Health insurance (yes versus no).
- Type of healthcare provider household members typically visit (public versus private).
- Wealth index (poorest, poorer, poor, richer, richest).
- Residence (rural versus urban).

Need factors.

- Number of the remaining major NCDs co-present.

All enabling variables except health insurance were measured at the household level. All predisposing and need factors except the number of household members were individual characteristics. Place of residence was the only variable not based on self-reported information. The wealth index is based on the ownership and type of household amenities such as flooring material, sanitation facilities, water sources, and electronic and transportation devices [35].

Several qualitative variables were collapsed into fewer categories before analysis (Supplemental Table 1). Hinduism is the most common religion in India, with Islam in a distant second [31]. Similar to previous studies, we grouped all other religions into a single category [36]. We grouped those not knowing their caste status with those not belonging to any of the backward/scheduled classes, assuming that those who do not know their caste status have not experienced any of the class-based marginalisation reflective of belonging to the backwards/scheduled classes. [37]. We grouped all non-public sectors into ‘private’ for the type of healthcare provider household members typically visit due to few respondents reporting a non-governmental organisation/trust hospital or other source (i.e., shop, home treatment, other) as their usual source of healthcare [36]. We grouped marital status congruently with the NFHS-5 report [30].

### Statistical analysis

Weighted descriptive statistics were first computed to summarise the sample characteristics. Quantitative variables were presented with median, interquartile range, and minimum and maximum. Qualitative variables were presented with raw counts and weighted prevalence estimates. Morbidity patterns were then calculated and presented graphically. Moreover, the number of individuals seeking treatment for their condition(s) was tabulated.

We then used survey-adjusted logistic regression to model the association between independent and dependent variables [38, 39]. We used the same independent variables for each NCD, with the most privileged or prevalent group as the qualitative variables’ reference category. There was no missing data. We reported the results as odds ratios (ORs) and adjusted odds ratios (aORs) with 95% confidence intervals (CI) [40]. We assessed multicollinearity by computing the adjusted generalised variance inflation factor [41]. Since none of the variables had a squared value greater than five, we deemed multicollinearity non-problematic [42, 43].

The NFHS-5 datasets come with survey weights attached. These weights are based on the product of the inverse of the individual’s response rate and the inverse of the household selection probability multiplied by the inverse household response rate [34]. Using these weights during analysis is important since they reduce bias in point estimates and measures of variability [34]. However, weights for men and women are calculated separately and do not account for the different sampling strategies (only men from 15% of [random] households were asked to participate). We, therefore, applied inverse probability re-weighting (1/0.15) to the men’s survey weights so that both men and women could be included in the same analysis.

We used R software version 4.1.2 for the analysis [44] with statistical significance set to $\alpha$ = 0.05.

## Results

### Descriptive data

Self-reported prevalence estimates for the four major NCDs ranged between 0.1% for cancer and 2.0% for diabetes. Differences in self-reported prevalence between men and women were the most noticeable for chronic respiratory disease, with women having about 0.5% points higher self-reported prevalence. Individuals with self-reported cancer were the youngest, and people with self-reported diabetes were the oldest. The formerly married, forward or casteless, and the insured had the highest self-reported prevalence across all four NCDs compared to the currently or never married, the backward classes, and the uninsured, respectively. The richest quintile only had the highest self-reported prevalence of diabetes (Table 1).

**Table 1﻿:** Characteristics of individuals aged 15–49 in India self-reporting the four major NCDs

Characteristic	Cancer n = 1 056 (0.1%)^1^	Chronic respiratory disease n = 10 534 (1.4%)^1^	Diabetes n = 13 501 (2.0%)^1^	Heart disease n = 5 861 (0.8%)^1^
Age				
Median (IQR)	35 (26, 44)	36 (28, 43)	41 (34, 46)	38 (28, 44)
Minimum–Maximum	15–49	15–49	15–49	15–49
Sex				
Male	136 (0.2%)	830 (1.2%)	1 595 (2.1%)	614 (0.9%)
Female	920 (0.1%)	9 704 (1.6%)	11 906 (1.9%)	5 247 (0.7%)
Marital status				
Currently married	787 (0.2%)	8 230 (1.7%)	11 470 (2.5%)	4 604 (1.0%)
Formerly married	66 (0.2%)	706 (2.5%)	954 (3.6%)	387 (1.3%)
Never married	203 (0.1%)	1 598 (0.8%)	1 077 (0.6%)	870 (0.4%)
Caste				
Forward caste or casteless	233 (0.2%)	2 881 (1.7%)	4 193 (2.5%)	1 768 (1.0%)
Other backward class	416 (0.1%)	3 838 (1.3%)	4 996 (1.9%)	1 937 (0.7%)
Scheduled tribe	223 (0.2%)	1 838 (1.3%)	1 781 (1.3%)	1 080 (0.8%)
Scheduled caste	184 (0.1%)	1 977 (1.4%)	2 531 (1.8%)	1 076 (0.8%)
Number of household members				
Median (IQR)	5 (4, 7)	4 (4, 6)	4 (3, 6)	5 (4, 6)
Minimum–Maximum	1–18	1–25	1–26	1–21
Religion				
Hindu	760 (0.1%)	7 586 (1.4%)	9 571 (2.0%)	3 807 (0.8%)
Muslim	150 (0.2%)	1 357 (1.5%)	2 211 (1.9%)	1 181 (1.2%)
Other	146 (< 0.1%)	1 591 (1.7%)	1 719 (2.4%)	873 (0.7%)
Years of education				
Median (IQR)	8 (4, 11)	8 (2, 10)	9 (4, 12)	7 (1, 10)
Minimum–Maximum	0–20	0–20	0–20	0–20
Has health insurance				
Yes	436 (0.2%)	4 197 (1.6%)	4 878 (2.4%)	2 006 (0.9%)
No	620 (0.1%)	6 337 (1.3%)	8 623 (1.8%)	3 855 (0.8%)
Where household members usually go for treatment				
Private	406 (0.1%)	4 224 (1.2%)	5 590 (2.0%)	2 226 (0.7%)
Public	650 (0.2%)	6 310 (1.7%)	7 911 (2.0%)	3 635 (0.9%)
Wealth index				
Richest	161 (0.1%)	1 729 (1.1%)	3 413 (2.9%)	835 (0.6%)
Richer	202 (0.2%)	2 137 (1.4%)	3 425 (2.4%)	1 120 (0.7%)
Middle	238 (0.2%)	2 327 (1.6%)	2 827 (1.9%)	1 297 (0.8%)
Poorer	263 (0.2%)	2 330 (1.6%)	2 193 (1.4%)	1 445 (1.0%)
Poorest	192 (0.1%)	2 011 (1.5%)	1 643 (1.1%)	1 164 (1.0%)
Residence				
Urban	254 (0.1%)	2 882 (1.4%)	4 721 (2.5%)	1 407 (0.7%)
Rural	802 (0.1%)	7 652 (1.5%)	8 780 (1.7%)	4 454 (0.9%)

^1^All n are the actual (unweighted) observations. Percentages are weighted and represent the national prevalence of self-reported disease

﻿IQR = Interquartile range

**Fig. 2:**
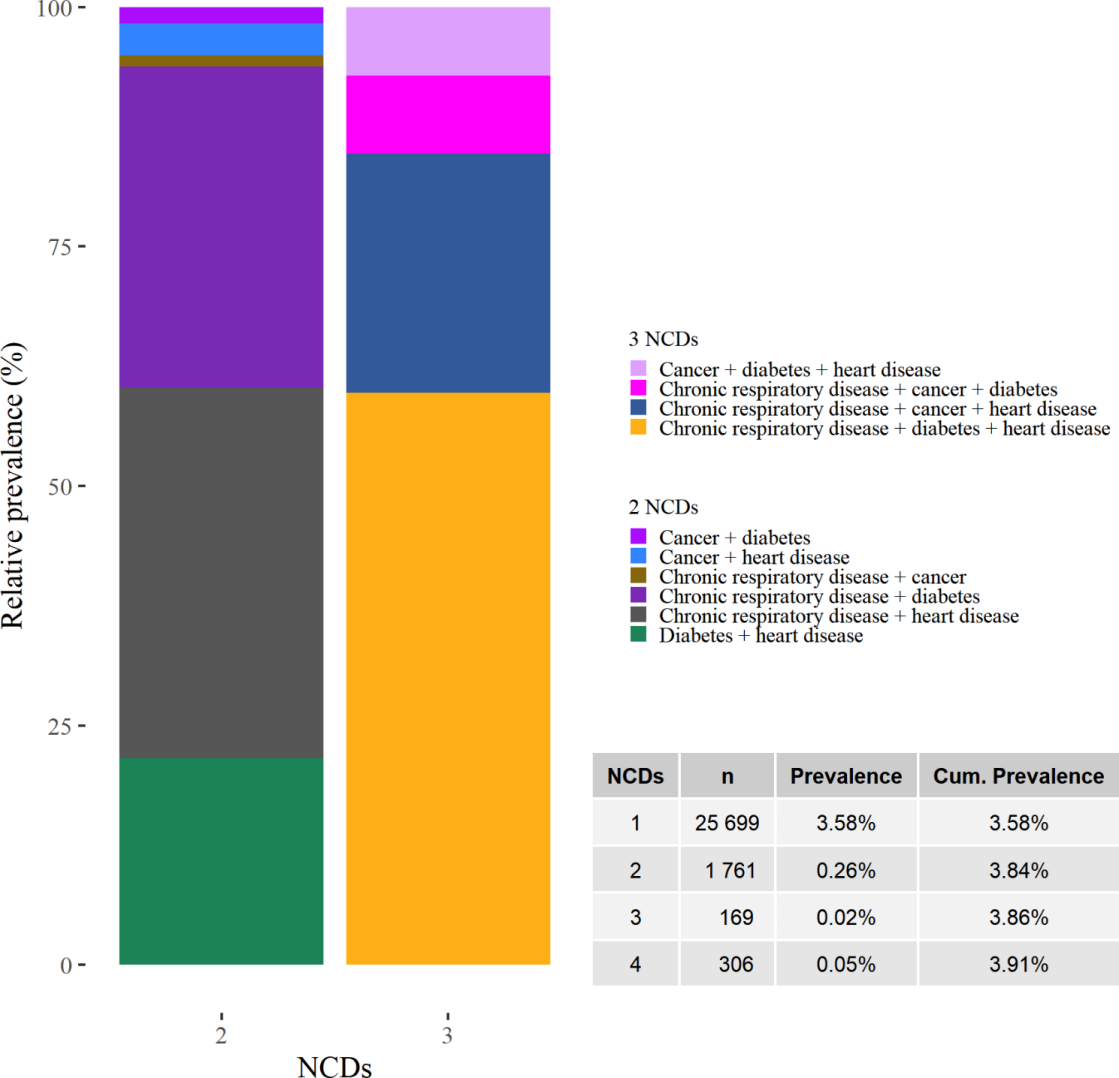
Self-reported morbidity counts, dyads and triads for the four major NCDs in India among adults aged 15–49. All n are the actual (unweighted) observations. Absolute and relative prevalence estimates have been weighted to be nationally representative. NCDs = Non-communicable diseases

The percentage of adults aged 15–49 in India self-reporting ≥ 1 of the four main NCDs was 3.9%. The most commonly reported morbidity dyads were chronic respiratory disease with coexisting heart disease or diabetes. The most commonly reported morbidity triad included the same three conditions (Fig. 2).

#### Outcome data

Treatment-seeking status differed depending on the condition: among those self-reporting a major NCD, the highest percentage who had sought treatment was observed for cancer (82%), whereas the lowest rate was observed for chronic respiratory disease (68%) (Table 2).

**Table 2:** Treatment-seeking status for the four major NCDs among individuals aged 15–49 with self-reported disease in India

	Has sought treatment for the disease^1^	
Condition	n	% (95% CI)
Cancer (n = 1 056)	846	82.3% (76.6–87%)
Chronic respiratory disease (n = 10 534)	7 176	67.7% (65.1–70%)
Diabetes (n = 13 501)	10 361	76.3% (74.1–78%)
Heart disease (n = 5 861)	4 307	74.2% (70.5–78%)

^1^All n are the actual (unweighted) observations. Percentages are weighted and represent national estimates of seeking treatment among those with self-reported disease

#### Regression analysis

##### Cancer

Holding all other variables constant, being Muslim (aOR: 3.44, 95% CI: 1.26–9.4), uninsured (aOR: 0.47, 95% CI: 0.24–0.93), never married (aOR: 2.97, 95% CI: 1.16–7.59) or belonging to the middle wealth quintile (aOR: 0.27, 95% CI: 0.08–0.94) was significantly associated with cancer treatment-seeking status among aware cases when compared to Hindus, the insured, those currently married, and the wealthiest 20%, respectively (Table 3).

**﻿Table 3:** Factors associated with seeking treatment for the four major NCDs among individuals aged 15–49 with self-reported disease in India

	Cancer		Chronic respiratory disease		Diabetes		Heart disease	
Factor	OR (95% CI)	aOR (95% CI)	OR (95% CI)	aOR (95% CI)	OR (95% CI)	aOR (95% CI)	OR (95% CI)	aOR (95% CI)
**Predisposing factors**								
Age	1.02 (0.99, 1.05)	1.08*** (1.04, 1.12)	1.01 (0.99, 1.02)	1.01* (1.00, 1.03)	1.06*** (1.05, 1.07)	1.05*** (1.03, 1.07)	1.03*** (1.02, 1.05)	1.05*** (1.02, 1.07)
Sex								
Male	—	—	—	—	—	—	—	—
Female	0.94 (0.48, 1.84)	1.33 (0.69, 2.57)	1.26 (0.96, 1.66)	1.35* (1.03, 1.76)	1.68*** (1.34, 2.09)	1.62*** (1.25, 2.11)	1.10 (0.77, 1.57)	1.15 (0.82, 1.63)
Marital status								
Currently married	—	—	—	—	—	—	—	—
Formerly married	0.85 (0.38, 1.91)	0.58 (0.22, 1.50)	0.93 (0.64, 1.36)	0.84 (0.60, 1.18)	1.16 (0.78, 1.73)	0.95 (0.63, 1.44)	1.01 (0.66, 1.56)	1.01 (0.63, 1.63)
Never married	1.08 (0.52, 2.27)	2.97* (1.16, 7.59)	1.09 (0.78, 1.54)	1.33 (0.90, 1.97)	0.29*** (0.21, 0.41)	0.69 (0.43, 1.10)	0.80 (0.49, 1.29)	1.56 (0.90, 2.70)
Caste								
Forward caste or casteless	—	—	—	—	—	—	—	—
Other backward class	0.46 (0.18, 1.20)	0.73 (0.26, 2.05)	1.01 (0.72, 1.40)	1.04 (0.77, 1.40)	0.77 (0.56, 1.05)	0.80 (0.58, 1.10)	1.02 (0.63, 1.65)	0.94 (0.60, 1.48)
Scheduled tribe	1.05 (0.31, 3.54)	2.00 (0.57, 7.09)	0.72 (0.47, 1.11)	0.95 (0.61, 1.48)	0.53* (0.33, 0.86)	0.71 (0.47, 1.07)	0.66 (0.37, 1.17)	0.81 (0.45, 1.43)
Scheduled caste	0.49 (0.17, 1.42)	1.08 (0.36, 3.23)	0.78 (0.52, 1.16)	0.85 (0.56, 1.27)	1.00 (0.70, 1.43)	1.25 (0.86, 1.81)	1.33 (0.76, 2.30)	1.38 (0.79, 2.39)
Number of household members	0.95 (0.84, 1.06)	0.89 (0.77, 1.03)	1.00 (0.95, 1.05)	1.00 (0.95, 1.05)	0.96 (0.92, 1.00)	0.98 (0.93, 1.04)	1.01 (0.95, 1.07)	1.05 (0.98, 1.12)
Religion								
Hindu	—	—	—	—	—	—	—	—
Muslim	1.95 (0.92, 4.16)	3.44* (1.26, 9.40)	1.14 (0.83, 1.57)	1.21 (0.85, 1.71)	1.12 (0.80, 1.56)	1.22 (0.86, 1.71)	0.75 (0.43, 1.31)	1.06 (0.61, 1.83)
Other	1.17 (0.44, 3.13)	1.91 (0.38, 9.50)	0.93 (0.65, 1.34)	0.96 (0.68, 1.35)	1.52 (0.90, 2.57)	1.18 (0.67, 2.06)	0.72 (0.44, 1.16)	0.67 (0.41, 1.10)
Years of education	1.05 (1.00, 1.10)	1.04 (0.97, 1.11)	1.02 (1.00, 1.04)	1.00 (0.98, 1.03)	1.00 (0.98, 1.02)	1.02 (0.99, 1.04)	1.03 (1.0, 1.06)	1.03 (0.99, 1.07)
**Enabling factors**								
Has health insurance								
Yes	—	—	—	—	—	—	—	—
No	0.67 (0.32, 1.41)	0.47* (0.24, 0.93)	0.99 (0.78, 1.27)	1.00 (0.79, 1.28)	0.88 (0.69, 1.12)	0.90 (0.71, 1.15)	0.75 (0.54, 1.04)	0.84 (0.58, 1.20)
Where household members usually go for treatment when sick								
Private	—	—	—	—	—	—	—	—
Public	1.02 (0.50, 2.10)	1.04 (0.55, 1.97)	0.95 (0.75, 1.22)	1.00 (0.80, 1.25)	0.99 (0.78, 1.25)	1.05 (0.83, 1.33)	0.76 (0.53, 1.08)	0.81 (0.59, 1.11)
Wealth index								
Richest	—	—	—	—	—	—	—	—
Richer	0.63 (0.22, 1.76)	0.81 (0.30, 2.18)	0.64* (0.43, 0.97)	0.72 (0.49, 1.07)	0.73 (0.48, 1.11)	0.74 (0.48, 1.14)	0.68 (0.33, 1.37)	0.74 (0.39, 1.38)
Middle	0.19** (0.06, 0.57)	0.27* (0.08, 0.94)	0.86 (0.60, 1.24)	1.04 (0.69, 1.57)	0.56** (0.38, 0.84)	0.65* (0.42, 1.00)	0.46* (0.23, 0.94)	0.62 (0.31, 1.25)
Poorer	0.25* (0.09, 0.72)	0.45 (0.14, 1.44)	0.52** (0.33, 0.81)	0.63 (0.35, 1.14)	0.40*** (0.26, 0.60)	0.45*** (0.28, 0.72)	0.34** (0.16, 0.71)	0.46* (0.24, 0.92)
Poorest	0.22** (0.07, 0.68)	0.36 (0.11, 1.19)	0.44*** (0.31, 0.64)	0.57* (0.36, 0.91)	0.37*** (0.23, 0.59)	0.42** (0.25, 0.73)	0.40** (0.20, 0.78)	0.59 (0.28, 1.22)
Residence								
Urban	—	—	—	—	—	—	—	—
Rural	0.40* (0.18, 0.89)	0.78 (0.30, 1.98)	0.66* (0.47, 0.91)	0.79 (0.55, 1.14)	0.65** (0.50, 0.86)	0.98 (0.75, 1.28)	0.67 (0.44, 1.01)	0.94 (0.61, 1.43)
**Need factors**								
NCDs	1.77*** (1.34, 2.32)	2.34*** (1.78, 3.07)	1.38*** (1.16, 1.65)	1.42*** (1.19, 1.71)	1.46*** (1.19, 1.78)	1.82*** (1.48, 2.24)	1.46*** (1.20, 1.77)	1.44*** (1.20, 1.74)

*p < 0.05; **p < 0.01; ***p < 0.001

OR = Odds Ratio, aOR = Adjusted Odds Ratio, CI = Confidence Interval

Furthermore, older individuals self-reporting cancer had 1.08 (95% CI: 1.04–1.12) times the odds of ever seeking treatment per year lived, whereas each additional condition out of the remaining three major NCDs was associated with 2.34 (95% CI: 1.78–3.07) times the odds of seeking cancer treatment.

##### Chronic Respiratory Disease

Among those who self-reported chronic respiratory disease, belonging to the poorest wealth quintile (aOR: 0.57, 95% CI: 0.36–0.91) was significantly associated with lower odds of seeking disease-specific treatment than the most affluent quintile. By contrast, being older (aOR: 1.01, 95% CI: 1–1.03 [per year]), female (aOR: 1.35, 95% CI: 1.03–1.76), or having other major NCDs (aOR: 1.42, 95% CI: 1.19–1.71 [per NCD]) was associated with increased odds of seeking treatment for chronic respiratory disease (Table 3).

##### Diabetes

Female (aOR: 1.62, 95% CI: 1.25–2.11), older (aOR: 1.05, 95% CI: 1.03–1.07 [per year]), and multimorbid (aOR: 1.82, 95% CI: 1.48–2.24 [per NCD]) people self-reporting diabetes were at increased odds of ever seeking diabetes treatment. By contrast, individuals self-reporting diabetes belonging to the middle (aOR: 0.65, 95% CI: 0.42–1) or lower ([aOR: 0.45, 95% CI: 0.28–0.72] [aOR: 0.43, 95% CI: 0.25–0.73] for the poorer and poorest, respectively) wealth quintiles were at significantly lower odds than the wealthiest of ever seeking diabetes treatment (Table 3).

##### Heart Disease

When adjusting for all other included covariates for individuals knowingly living with heart disease, age (aOR: 1.05, 95% CI: 1.02–1.07), coexisting major NCDs (aOR: 1.44, 95% CI: 1.2–1.74 [per NCD]) and belonging to the poorer wealth quintile (aOR: 0.46, 95% CI: 0.24–0.92) were significantly associated with treatment-seeking status (Table 3).

## Discussion

To the best of our knowledge, this is the first study which has attempted to systematically investigate the factors associated with seeking treatment for the four main NCDs among aware cases in India. Developing a better understanding of treatment-seeking behaviours for these NCDs is needed since improvements in the access to and delivery of treatment represent an essential component in addressing India’s avertable NCD mortality [3, 4]. The need to develop a better understanding of the factors associated with treatment-seeking behaviours for the four main NCDs is further underscored by the fact that the share of aware persons who have never sought treatment is alarmingly high, ranging from one-fifth among those who reported living with cancer to one-third among those self-reporting living with chronic respiratory disease.

We adopted the widely acknowledged Behavioural Model of Health Services Use [26–28] in our analysis of recent and nationally representative data, which helped uncover the predisposing, enabling, and need factors associated with seeking disease-specific treatment among 15–49-year-olds self-reporting any of the four main NCDs in India. A key finding of this study is that variables from all three overarching factor categories were significantly associated with treatment-seeking behaviour across the four NCDs. Greater age (a predisposing factor) and reporting multiple major NCDs (a need factor) were significantly associated with greater odds of seeking disease-specific treatment among aware cases. By contrast, individuals in the middle or lower wealth quintiles had lower odds of seeking treatment than the wealthiest 20%. These variables and overarching factor categories represent distinct constructs for explaining treatment-seeking behaviours and subsequent use, suggesting the need for a multi-pronged approach to optimise treatment uptake among those knowingly living with major NCDs in India.

### Predisposing and need factors associated with treatment-seeking status

Self-reported major NCD co-morbidities, the only need factor included in our study, were significantly associated with increased odds of seeking disease-specific treatment for each NCD, confirming the importance of considering need factors when studying treatment-seeking behaviours. The increased healthcare needs of people living with multiple chronic conditions in India are well documented. A 2015 systematic review established that Indians with numerous chronic conditions often experience psychological distress and lowered quality of life and physical capacity [45]. Our study adds to this evidence base by showcasing the role of co-morbid major NCDs in seeking treatment for the four main NCDs.

Greater age was also significantly associated with increased odds of seeking disease-specific treatment for all four NCDs among aware cases. The positive association between age and seeking treatment expands on previous research in India focusing on 15–49-year-olds with heart disease [24] and diabetes [25] and suggests that age is an important predisposing factor for explaining treatment-seeking behaviour across all four major NCDs. According to Andersen and Newman [27, 28], the increased propensity to use healthcare services among older individuals stems from increased illness across the life course. Since other co-existing diseases (besides the major NCDs, which we included as a need factor) become more prevalent with age [46–48], this explanation also seems plausible in the present context.

We also found that women were at greater odds of ever seeking treatment than men for all conditions; however, large statistical uncertainty surrounded all point estimates except for diabetes and chronic respiratory disease. Among aware cases, women had 1.62 and 1.35 times the odds of ever seeking treatment for diabetes and chronic respiratory disease, respectively. These findings might reflect biological differences in health status between the sexes [26–28], as previous studies have linked the female reproductive physiology to a higher risk of complications during diabetes and more severe symptoms during chronic respiratory disease [49, 50].

Although the aforementioned need and demographic predisposing factors are generally considered equitable sources of variation in healthcare access [26], we argue that such differences are only justifiable for the type or amount of treatment received – not whether any treatment is sought. Our findings suggest the need to encourage people with comparatively less illness to be more proactive in seeking out treatment for their NCDs, which could potentially be facilitated through educational campaigns outlining the benefits of seeking timely treatment.

Religion and marital status were the only predisposing factors significantly associated with seeking treatment in the present study. Muslims and the never-married self-reporting cancer had roughly three times the odds of seeking cancer treatment than Hindus and the currently married ones. The fact that the never-married had greater odds of seeking cancer treatment than the presently married is a finding to be noted, given that marriage is typically considered a source of social support that can be leveraged to access health services [51]. Studies focusing on participation in cancer screening in India have also found results conflicting with ours, including lower compliance with breast cancer screening among Muslims than Hindus and lower participation rates in cervical screening programmes among unmarried than married women [52, 53]. Therefore, further research is needed to better understand the relationship between religion, marital status, and seeking cancer treatment and provide more conclusive information that supports or contradicts our findings.

### Enabling factors associated with treatment-seeking status

Wealth was significantly associated with treatment-seeking status across the four main NCDs: the less affluent knowingly living with disease consistently showed lower odds of seeking treatment than the wealthiest quintile despite adjusting for predisposing, other enabling, and need factors. This finding is most likely reflective of India’s health financing system. Approximately half of the country’s health spending is financed through out-of-pocket expenditure [54]. For NCDs specifically, the costs incurred by patients are primarily for treatment [19]. Whereas the wealthiest may deal with such expenses by reducing non-essential expenditures, the poor must often borrow or sell assets [55]. However, borrowing or selling off assets is not a sustainable health financing solution for NCDs since these conditions often require prolonged treatment with recurring expenses [56]. Therefore, our study suggests that the less affluent are more likely to avoid seeking treatment altogether, possibly due to the anticipated financial burden of managing the major NCDs.

In contrast to household wealth, health insurance only uniquely contributed to seeking cancer treatment. Specifically, the insured had two times the odds of seeking cancer treatment than the uninsured. This may be due to the high costs associated with cancer treatment, which account for nearly 80% of all catastrophic health expenditures in India [57]. Although health insurance has not traditionally covered cancer treatment, coverage has become more common in government insurance schemes [58]. Our findings suggest that health insurance, whether through reimbursement for expenses or lump sum payments upon diagnosis, may play an essential role in facilitating access to cancer treatment, which is an expensive disease to manage. However, we note that respondents self-reporting cancer had varying types of health insurance (Supplementary Table 2), so further research is needed to fully understand the potential impact of health insurance coverage on cancer treatment-seeking behaviour.

### Limitations

Our study has some limitations that should be considered when interpreting the results. Firstly, since the NFHS is limited to 15–49-year-olds, results might not be generalisable to those for whom these NCDs are most common (i.e., adults aged 50 and above). Secondly, we focused on examining the treatment-seeking behaviour among those who self-reported and thus were aware of having any of the four main NCDs, as awareness represents an important catalyst in the healthcare access process [22]. Consequently, our results do not represent people unknowingly living with NCDs. Unaware cases constitute a substantial portion of all NCD cases: an analysis of NFHS-4 (2015–16), which complemented self-reports with blood glucose readings, found that almost half of this age group’s diabetes prevalence (which was 2.9% at the time) was unreported [33]. Previous research in India focusing on treatment-seeking behaviours for heart disease [24] and treatment utilisation patterns for diabetes [59] have employed Heckman-type selection models to generalise results beyond aware cases. However, the use of such models remains contested, as they rely on a theoretically valid exclusion restriction (i.e., a variable understood as being independently associated with self-reporting an NCD but not seeking or availing treatment for it) to produce unbiased estimates [60–63]. Thirdly, our study did not consider whether those who had sought treatment could procure it, nor the quality of treatment received. Ensuring the quality of care through appropriate management and retention is also necessary in minimising avoidable NCD mortality in LMICs [4]. Lastly, although we included 12 predisposing, enabling, and need factors in our analysis, it is not a fully exhaustive account. Occupation, for instance, is a common predisposing factor in Andersen’s model [26], but we could not include employment-related information since it was only collected in 15% of randomly selected households. We also did not consider that the studied factors may be intersecting and mutually reinforcing sources of marginalisation or privilege. Such interactions are at the centre of intersectional theory, which has been recognised as an important theoretical framework in public health [64]. Future research studying treatment-seeking behaviours for NCDs should therefore consider combining the behavioural model of health services use with intersectional theory due to the latter’s potential to document health inequalities more precisely [65]. Such research should ideally rely on a mixed methods approach to fully capture the reasons underlying treatment-seeking behaviours.

## Conclusion

In conclusion, the present study found that greater age and having multiple major NCDs co-present was associated with an increased likelihood of seeking disease-specific treatment for the four main NCDs among aware 15–49-year-old cases in India. By contrast, the less affluent who knowingly lived with any of the four main NCDs had lower odds of seeking disease-specific care. Associations between treatment-seeking status and other predisposing factors (religion, marital status, and sex) and enabling factors (insurance coverage) depended on the NCD. The fact that predisposing, enabling, and need factors were associated with treatment-seeking status suggests that health policies in India should take a multi-pronged approach to improving access to treatment among aware cases. The potential and need for improving access to and use of treatment services for the four main NCDs is further underscored by the fact that roughly one-fifth (cancer) to one-third (chronic respiratory disease) of 15–49-year-olds reporting a major NCD in India have never sought treatment, despite being aware of their condition.

### Electronic supplementary material

Below is the link to the electronic supplementary material.

Supplementary Material 1

## Data Availability

R Scripts used for data management and analysis are available upon request from the corresponding author. The NFHS-5 dataset is available upon request from the DHS website after registration: https://dhsprogram.com/data/available-datasets.cfm.

## Declarations

The survey (NFHS-5) protocol and questionnaires received ethical permission from the Institutional Review Board of the Indian Institute of Population Sciences (IIPS), Mumbai, India and the ICF Institutional Review Board. All methods were carried out per the ethical committee’s relevant guidelines and regulations. Data collectors were rigorously trained, and quality control was ensured. To comply with the ethical conditions, informed consent was obtained from the respondents. For under-aged respondents (< 18 years old), the data collectors received informed consent from a parent or an adult caregiver. The privacy of the respondents was guaranteed. More details of the ethical issues are available in the National Family Health Survey India Report 2019–2021.

## References

[1] Di Cesare M, Khang Y-H, Asaria P (2013). Inequalities in non-communicable diseases and effective responses. Lancet.

[2] Global Burden of Disease Collaborative Network. Global Burden of Disease Study 2019. (GBD 2019) results. Seattle, WA: Institute for Health Metrics and Evaluation (IHME); 2020. https://vizhub.healthdata.org/gbd-results/ (accessed 27 Jul 2023).

[3] Bennett JE, Kontis V, Mathers CD et al. NCD Countdown 2030: pathways to achieving Sustainable Development Goal target 3.4. Lancet. 2020;396:918–34. 10.1016/S0140-6736(20)31761-X. 10.1016/S0140-6736(20)31761-X PMC7470795 32891217

[4] Kruk ME, Gage AD, Joseph NT (2018). Mortality due to low-quality health systems in the universal health coverage era: a systematic analysis of amenable deaths in 137 countries. Lancet.

[5] Murray CJL, Abbafati C, Abbas KM (2020). Five insights from the Global Burden of Disease Study 2019. Lancet.

[6] Bloom DE, Cafiero-Fonseca ET, Candeias V (2014). Economics of non-communicable diseases in India: the costs and returns on investment of interventions to promote healthy living and prevent, treat, and manage NCDs.

[7] The World Bank. World Bank country and lending groups. https://datahelpdesk.worldbank.org/knowledgebase/articles/906519-world-bank-country-and-lending-groups (accessed 27 Jul 2023).

[8] United Nations Department of Economic and Social Affairs., Population Division. World population prospects 2022: summary of results. New York, NY: United Nations; 2022. UN DESA/POP/2022/TR/NO. 3.

[9] Webster AC, Nagler EV, Morton RL (2017). Chronic kidney disease. Lancet.

[10] Thakur JS, Paika R, Singh S (2020). Burden of noncommunicable diseases and implementation challenges of national NCD programmes in India. Med J Armed Forces India.

[11] Directorate General of Health Services., Ministry of Health and Family Welfare, Government of India. National Programme for Prevention and Control of Cancer, Diabetes, Cardiovascular Diseases and Stroke (NPCDCS), Operational Guidelines (Revised: 2013-17). 2013.

[12] Shroff ZC, Marten R, Ghaffar A (2020). On the path to universal health coverage: aligning ongoing health systems reforms in India. BMJ Glob Health.

[13] Lahariya C (2018). 'Ayushman Bharat’ program and universal health coverage in India. Indian Pediatr.

[14] Angell BJ, Prinja S, Gupt A (2019). The Ayushman Bharat Pradhan Mantri Jan Arogya Yojana and the path to universal health coverage in India: overcoming the challenges of stewardship and governance. PLoS Med.

[15] UN General Assembly. Transforming our world: the 2030 Agenda for Sustainable Development. United Nations; 2015. A/RES/70/1

[16] World Health Organization (2013). Global action plan for the prevention and control of noncommunicable diseases 2013–2020.

[17] Elias MA, Pati MK, Aivalli P (2018). Preparedness for delivering non-communicable disease services in primary care: access to medicines for diabetes and hypertension in a district in south India. BMJ Glob Health.

[18] Krishnan A, Mathur P, Kulothungan V (2021). Preparedness of primary and secondary health facilities in India to address major noncommunicable diseases: results of a National Noncommunicable Disease Monitoring Survey (NNMS). BMC Health Serv Res.

[19] Behera S, Pradhan J (2021). Uneven economic burden of non-communicable diseases among Indian households: a comparative analysis. PLoS ONE.

[20] Ward H, Mertens TE, Thomas C (1997). Health seeking behaviour and the control of sexually transmitted disease. Health Policy Plan.

[21] von Lengerke T, Gohl D, Babitsch B, Janssen C, Swart E, von Lengerke T (2014). Re-revisiting the behavioral model of health care utilization by Andersen: a review on theoretical advances and perspectives. Health care utilization in Germany: theory, methodology, and results.

[22] Ricketts TC, Goldsmith LJ (2005). Access in health services research: the battle of the frameworks. Nurs Outlook.

[23] Shaikh BT, Hatcher J (2005). Health seeking behaviour and health service utilization in Pakistan: challenging the policy makers. J Public Health Oxf Engl.

[24] Mishra R, Monica (2019). Determinants of cardiovascular disease and sequential decision-making for treatment among women: a Heckman’s approach. SSM Popul Health.

[25] Prenissl J, Jaacks LM, Mohan V (2019). Variation in health system performance for managing diabetes among states in India: a cross-sectional study of individuals aged 15 to 49 years. BMC Med.

[26] Andersen RM (1995). Revisiting the behavioral model and access to medical care: does it matter?. J Health Soc Behav.

[27] Andersen RM, Newman JF (2005). Societal and individual determinants of medical care utilization in the United States. Milbank Q.

[28] Andersen RM, Newman JF (1973). Societal and individual determinants of medical care utilization in the United States. Milbank Mem Fund Q Health Soc.

[29] Andersen RM (2008). National health surveys and the behavioral model of health services use. Med Care.

[30] International Institute for Population Sciences (IIPS), ICF (2022). National Family Health Survey (NFHS-5), 2019-21.

[31] Registrar General and Census Commissioner of India (2011). Census of India 2011.

[32] Ghosh S, Kumar M (2019). Prevalence and associated risk factors of hypertension among persons aged 15–49 in India: a cross-sectional study. BMJ Open.

[33] Claypool KT, Chung M-K, Deonarine A (2020). Characteristics of undiagnosed diabetes in men and women under the age of 50 years in the Indian subcontinent: the National Family Health Survey (NFHS-4)/Demographic Health Survey 2015–2016. BMJ Open Diabetes Res Care.

[34] Croft TN, Marshall AM, Allen CK. Guide to DHS statistics. Rockville, MD: ICF; 2018. https://www.dhsprogram.com/pubs/pdf/DHSG1/Guide_to_DHS_Statistics_DHS-7.pdf (accessed 27 Jul 2023).

[35] ICF. Wealth Index construction. The DHS Program website funded by USAID. https://dhsprogram.com/topics/wealth-index/Wealth-Index-Construction.cfm (accessed 27 Jul 2023).

[36] Thind A, Mohani A, Banerjee K (2008). Where to deliver? Analysis of choice of delivery location from a national survey in India. BMC Public Health.

[37] Nayar KR (2007). Social exclusion, caste & health: a review based on the social determinants framework. Indian J Med Res.

[38] Lumley T (2004). Analysis of complex survey samples. J Stat Softw.

[39] Lumley T. survey: analysis of complex survey samples. 2020.

[40] Vandenbroucke JP, von Elm E, Altman DG (2007). Strengthening the reporting of observational studies in epidemiology (STROBE): explanation and elaboration. Ann Intern Med.

[41] Fox J, Monette G (1992). Generalized collinearity diagnostics. J Am Stat Assoc.

[42] Fox J. Applied regression analysis and generalized linear models. 3rd ed. Thousand Oaks, CA: Sage Publications; 2015.

[43] James G, Witten D, Hastie T et al. An introduction to statistical learning. 2nd ed. New York, NY: Springer; 2021. https://www.statlearning.com/ (accessed 27 Jul 2023).

[44] R Core Team. R: A language and environment for statistical computing. 2021. https://www.R-project.org/.

[45] Pati S, Swain S, Hussain MA (2015). Prevalence and outcomes of multimorbidity in South Asia: a systematic review. BMJ Open.

[46] Davis JW, Chung R, Juarez DT (2011). Prevalence of Comorbid conditions with aging among patients with diabetes and cardiovascular disease. Hawaii Med J.

[47] Barnett K, Mercer SW, Norbury M (2012). Epidemiology of multimorbidity and implications for health care, research, and medical education: a cross-sectional study. Lancet.

[48] Veenendaal M, Westerik JAM, van den Bemt L (2019). Age- and sex-specific prevalence of chronic comorbidity in adult patients with asthma: a real-life study. NPJ Prim Care Respir Med.

[49] Kautzky-Willer A, Harreiter J, Pacini G (2016). Sex and gender differences in risk, pathophysiology and complications of type 2 diabetes mellitus. Endocr Rev.

[50] Somayaji R, Chalmers JD. Just breathe: a review of sex and gender in chronic lung disease. Eur Respir Rev. 2022;31. 10.1183/16000617.0111-2021. 10.1183/16000617.0111-2021 PMC9488531 35022256

[51] Pandey KR, Yang F, Cagney KA (2019). The impact of marital status on health care utilization among Medicare beneficiaries. Medicine (Baltimore).

[52] Kulkarni SV, Mishra GA, Dusane RR (2019). Determinants of compliance to breast cancer screening and referral in low socio-economic regions of urban India. Int J Prev Med.

[53] Nene B, Jayant K, Arrossi S (2007). Determinants of women’s participation in cervical cancer screening trial, Maharashtra, India. Bull World Health Organ.

[54] National Health Systems Resource Centre. National health accounts estimates for India 2017–18. New Delhi, India: Ministry of Health and Family Welfare, Government of India; 2021.

[55] Bhojani U, Thriveni B, Devadasan R (2012). Out-of-pocket healthcare payments on chronic conditions impoverish urban poor in Bangalore, India. BMC Public Health.

[56] Ewen M, Zweekhorst M, Regeer B (2017). Baseline assessment of WHO’s target for both availability and affordability of essential medicines to treat non-communicable diseases. PLoS ONE.

[57] Kastor A, Mohanty SK (2018). Disease-specific out-of-pocket and catastrophic health expenditure on hospitalization in India: do Indian households face distress health financing?. PLoS ONE.

[58] Pramesh CS, Badwe RA, Borthakur BB (2014). Delivery of affordable and equitable cancer care in India. Lancet Oncol.

[59] Barman P, Das M, Verma M (2023). Epidemiology of type 2 diabetes mellitus and treatment utilization patterns among the elderly from the first wave of Longitudinal Aging Study in India (2017-18) using a Heckman selection model. BMC Public Health.

[60] Wolfolds SE, Siegel J (2019). Misaccounting for endogeneity: the peril of relying on the Heckman two-step method without a valid instrument. Strateg Manag J.

[61] Lennox CS, Francis JR, Wang Z (2012). Selection models in accounting research. Acc Rev.

[62] Bushway S, Johnson BD, Slocum LA (2007). Is the magic still there? The use of the Heckman two-step correction for selection bias in criminology. J Quant Criminol.

[63] Puhani P (2000). The Heckman correction for sample selection and its critique. J Econ Surv.

[64] Bowleg L (2012). The problem with the phrase women and minorities: intersectionality—an important theoretical framework for public health. Am J Public Health.

[65] Bauer GR (2014). Incorporating intersectionality theory into population health research methodology: challenges and the potential to advance health equity. Soc Sci Med.

