# Supplementary material for: Treatment-seeking behaviour among 15–49-year-olds with self-reported heart disease, cancer, chronic respiratory disease, and diabetes: a national cross-sectional study in India
Source: BMC Public Health. 2023 Nov 8;23:2197. doi: 10.1186/s12889-023-17123-3 (PMC10631191; doi:10.1186/s12889-023-17123-3)
Supplement: Supplementary file 1 — Supplementary Material 1 [file 12889_2023_17123_MOESM1_ESM.docx]

| **Supplementary Table 1.** Original and collapsed categories | | | | | |
| --- | --- | --- | --- | --- | --- |
|  |  | Cancer | Chronic respiratory disease | Diabetes | Heart disease |
| Predisposing | | | | | |
| **Caste** | | | | | |
|  | **Scheduled caste** | **184 (18.5)** | **1977 (20.8)** | **2531 (19.0)** | **1076 (21.4)** |
|  | **Scheduled tribe** | **223 (10.5)** | **1838 (8.6)** | **1781 (6.2)** | **1080 (9.0)** |
|  | **Other backward class** | **416 (40.5)** | **3838 (37.4)** | **4996 (40.2)** | **1937 (35.9)** |
|  | **Forward caste or casteless** | **233 (30.5)** | **2881 (33.3)** | **4193 (34.7)** | **1768 (33.7)** |
|  | None of them | 157 (16.4) | 2068 (21.7) | 3076 (24.7) | 1184 (18.5) |
|  | Don't know | 6 (0.4) | 78 (1.3) | 83 (1.1) | 35 (1.4) |
|  | No caste/tribe | 70 (13.7) | 735 (10.3) | 1034 (8.8) | 549 (13.8) |
| **Marital status** | | | | | |
|  | **Never married** | **203 (20.0)** | **1598 (18.3)** | **1077 (9.8)** | **870 (15.6)** |
|  | Never married | 200 (19.8) | 1584 (18.1) | 1064 (9.7) | 866 (15.5) |
|  | Married, gauna not performed | 3 (0.2) | 14 (0.1) | 13 (0.1) | 4 (0.1) |
|  | **Currently married** | **787 (76.4)** | **8230 (76.8)** | **11470 (84.9)** | **4604 (79.8)** |
|  | **Formerly married** | **66 (3.7)** | **706 (4.9)** | **954 (5.3)** | **387 (4.7)** |
|  | Widowed | 45 (2.4) | 500 (3.3) | 757 (3.9) | 285 (3.2) |
|  | Divorced | 9 (0.4) | 79 (0.8) | 75 (0.4) | 39 (0.6) |
|  | Separated | 10 (0.8) | 98 (0.6) | 95 (0.9) | 50 (0.7) |
|  | Deserted | 2 (0.1) | 29 (0.2) | 27 (0.1) | 13 (0.1) |
| **Religion** | | | | | |
|  | **Hindu** | **760 (77.3)** | **7586 (79.0)** | **9571 (79.7)** | **3807 (74.7)** |
|  | **Muslim** | **150 (19.6)** | **1357 (15.0)** | **2211 (13.9)** | **1181 (21.0)** |
|  | **Other** | **146 (3.1)** | **1591 (6.1)** | **1719 (6.3)** | **873 (4.2)** |
|  | Christian | 86 (1.1) | 1072 (3.7) | 937 (2.9) | 514 (2.2) |
|  | Sikh | 22 (1.0) | 243 (1.0) | 422 (1.9) | 185 (1.2) |
|  | Buddhist/Neo-Buddhist | 17 (0.8) | 119 (0.8) | 190 (0.5) | 93 (0.4) |
|  | Jain | 1 (0.0) | 24 (0.2) | 22 (0.3) | 4 (0.0) |
|  | Jewish | 0 (0.0) | 0 (0.0) | 0 (0.0) | 0 (0.0) |
|  | Parsi/Zoroastrian | 0 (0.0) | 2 (0.0) | 2 (0.0) | 1 (0.0) |
|  | No religion | 0 (0.0) | 4 (0.0) | 2 (0.6) | 0 (0.0) |
|  | Other | 20 (0.2) | 127 (0.3) | 144 (0.1) | 76 (0.4) |

| **Supplementary Table 1 (continued).** Original and collapsed categories | | | | | |
| --- | --- | --- | --- | --- | --- |
|  | | Cancer | Chronic respiratory disease | Diabetes | Heart disease |
| Enabling | | | | | |
| **Where household members usually go for treatment when sick** | | | | | |
|  | **Public** | **650 (54.0)** | **6310 (58.0)** | **7911 (49.3)** | **3635 (55.7)** |
|  | Public: Government/municipal hospital | 288 (21.8) | 2862 (25.0) | 3684 (23.7) | 1648 (22.7) |
|  | Public: Government dispensary | 34 (2.0) | 288 (2.3) | 338 (1.8) | 164 (1.6) |
|  | Public: UHC/UHP/UFWC | 12 (1.8) | 111 (1.1) | 162 (1.3) | 55 (1.0) |
|  | Public: CHC/rural hospital/block PHC | 157 (12.5) | 1697 (16.1) | 1860 (12.0) | 931 (18.6) |
|  | Public: PHC/additional PHC | 126 (14.1) | 1147 (11.6) | 1522 (9.1) | 639 (9.6) |
|  | Public: Sub-centre | 29 (1.8) | 143 (1.0) | 288 (1.2) | 153 (1.6) |
|  | Public AYUSH: AYURVEDA | 0 (0.0) | 5 (0.0) | 7 (0.0) | 3 (0.0) |
|  | Public AYUSH: YOGA AND NATUROPATHY | 0 (0.0) | 0 (0.0) | 2 (0.0) | 0 (0.0) |
|  | Public AYUSH: UNANI | 0 (0.0) | 0 (0.0) | 1 (0.0) | 0 (0.0) |
|  | Public AYUSH: SIDDHA | 0 (0.0) | 0 (0.0) | 0 (0.0) | 0 (0.0) |
|  | Public AYUSH: HOMEOPATHY | 0 (0.0) | 13 (0.1) | 10 (0.1) | 9 (0.2) |
|  | Public AYUSH: SOWA RIGPA (TTM) | 1 (0.0) | 3 (0.0) | 4 (0.0) | 4 (0.0) |
|  | Public AYUSH: OTHER | 0 (0.0) | 1 (0.1) | 0 (0.0) | 1 (0.0) |
|  | Public: ANGANWADI/ICDS centre | 1 (0.0) | 1 (0.0) | 3 (0.0) | 2 (0.0) |
|  | Public: ASHA | 0 (0.0) | 7 (0.1) | 2 (0.0) | 2 (0.0) |
|  | Public: Government mobile clinic | 2 (0.1) | 10 (0.1) | 11 (0.0) | 14 (0.1) |
|  | Other public sector | 0 (0.0) | 22 (0.5) | 17 (0.1) | 10 (0.2) |
|  | **Private** | **406 (46.0)** | **4224 (42.0)** | **5590 (50.7)** | **2226 (44.3)** |
|  | NGO or trust hospital/clinic | 2 (0.1) | 33 (1.0) | 50 (0.4) | 17 (0.4) |
|  | Private hospital | 188 (20.2) | 1728 (15.5) | 2564 (23.1) | 848 (16.6) |
|  | Private doctor/clinic | 184 (21.3) | 2132 (21.5) | 2613 (24.1) | 1167 (23.5) |
|  | Private paramedic) | 5 (1.3) | 38 (0.3) | 58 (0.3) | 17 (0.4) |
|  | Private AYUSH: AYURVEDA | 0 (0.0) | 6 (0.0) | 1 (0.0) | 1 (0.0) |
|  | Private AYUSH: YOGA AND NATUROPATHY | 2 (0.1) | 1 (0.0) | 0 (0.0) | 1 (0.0) |
|  | Private AYUSH: UNANI | 1 (0.0) | 2 (0.0) | 1 (0.0) | 0 (0.0) |
|  | Private AYUSH: SIDDHA | 0 (0.0) | 0 (0.0) | 0 (0.0) | 0 (0.0) |
|  | Private AYUSH: HOMEOPATHY | 1 (0.1) | 7 (0.0) | 4 (0.0) | 3 (0.1) |
|  | Private AYUSH: SOWA RIGPA (TTM) | 0 (0.0) | 3 (0.0) | 1 (0.0) | 0 (0.0) |
|  | Private AYUSH: OTHER | 0 (0.0) | 0 (0.0) | 1 (0.0) | 1 (0.0) |
|  | Private: Traditional healer | 0 (0.0) | 10 (0.1) | 11 (0.1) | 3 (0.1) |
|  | Private: Pharmacy/drugstore | 7 (0.8) | 85 (0.9) | 81 (0.6) | 45 (0.4) |
|  | Private: DAI (TBA) | 0 (0.0) | 2 (0.0) | 2 (0.0) | 0 (0.0) |
|  | Other private health sector | 12 (1.7) | 70 (0.7) | 86 (0.7) | 61 (1.6) |
|  | Shop | 0 (0.0) | 15 (0.2) | 16 (0.1) | 10 (0.1) |
|  | Home treatment | 0 (0.0) | 10 (0.1) | 16 (0.2) | 6 (0.1) |
|  | Other | 4 (0.5) | 82 (1.6) | 85 (1.1) | 46 (1.0) |
| Note: all frequencies are unweighted counts among de jure 15–49-year-old respondents self-reporting a particular NCD. Percentages within parentheses are weighted to be nationally representative and are calculated column-wise. | | | | | |

| **Supplementary Table 2.** Share of respondents with a particular type of health insurance among 15-49-year-olds self-reporting major NCDs | | | | |
| --- | --- | --- | --- | --- |
|  | Cancer | Chronic respiratory disease | Diabetes | Heart disease |
| Employee State Insurance Scheme | 3.7 | 1.2 | 2.7 | 1.7 |
| Central Government Health Scheme | 4.0 | 1.7 | 2.8 | 1.8 |
| State Health Insurance Scheme | 15.6 | 19.1 | 18.7 | 17.9 |
| Rashtriya Swasthya Bima Yojana | 8.1 | 6.4 | 6.7 | 5.4 |
| Community Health Insurance Programme | 0.2 | 0.2 | 0.1 | 0.2 |
| Other Health Insurance Through Employer | 0.1 | 0.2 | 0.4 | 0.7 |
| Medical Reimbursement from Employer | 0.1 | 0.2 | 0.4 | 0.2 |
| Other Privately Purchased Commercial Health Insurance | 0.1 | 1.6 | 1.8 | 1.2 |
| Other | 8.8 | 6.9 | 7.1 | 7.7 |
| Note: respondents could select more than one health insurance scheme. Percentages are weighted to be nationally representative. | | | | |
